# Supplementary material for: Neural mechanisms underlying reward processing and social cognition: A replication study with a Japanese sample
Source: PLoS One. 2025 Oct 22;20(10):e0328424. doi: 10.1371/journal.pone.0328424 (PMC12543148; doi:10.1371/journal.pone.0328424)
Supplement: S1 Fig — (PDF) [file pone.0328424.s001.pdf]

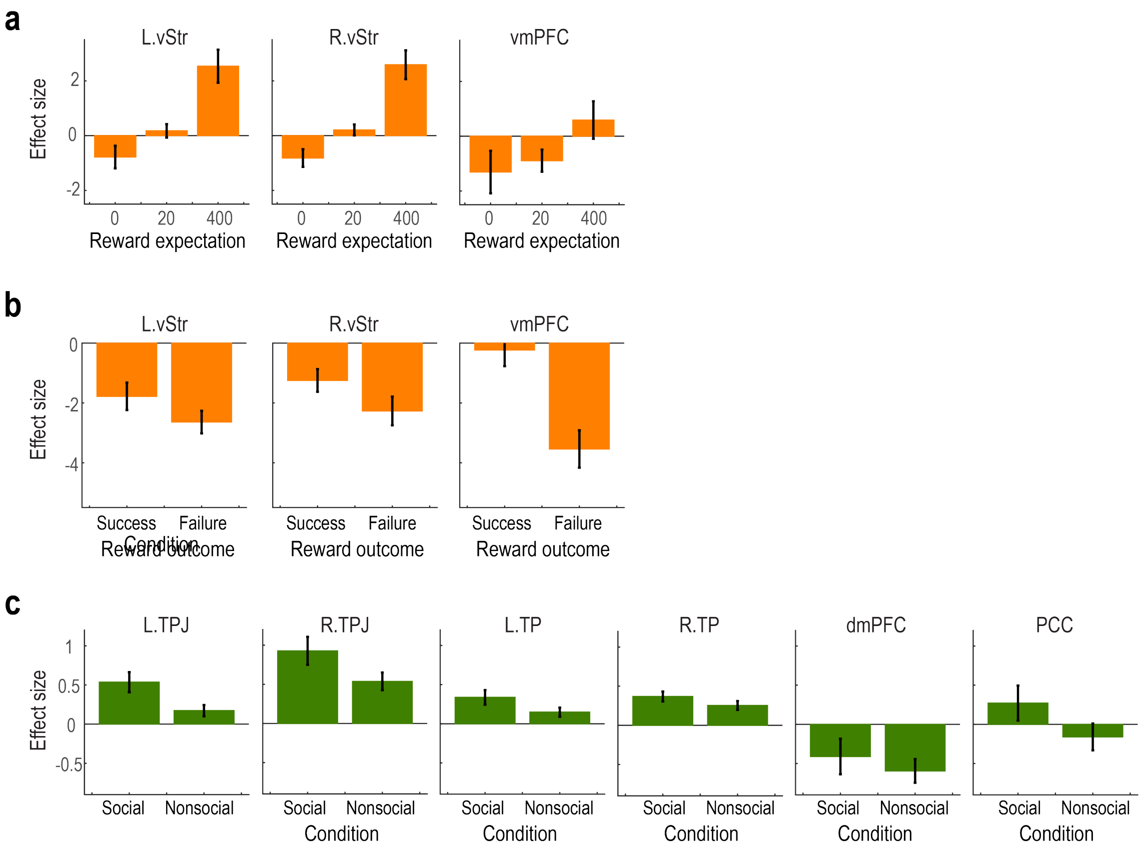

Figure S1: Supplementary analysis on the neuroimaging data I.

(a) Independent ROI analysis of reward expectation in the MID task. Effect sizes (signal changes from baseline) in the Cue phase are plotted as a function of reward expectation (i.e., the monetary amount available upon a successful outcome) (Mean ± SEM across participants). L.vStr, left ventral striatum; R.vStr, right ventral striatum; and vmPFC, ventromedial prefrontal cortex.

(b) Independent ROI analysis of reward outcome in the MID task. Effect sizes (signal changes from baseline) in the Outcome phase are shown separately for success and failure outcomes (Mean ± SEM across participants).

(c) Independent ROI analysis of social cognition in the ToM task. Effect sizes (signal changes from baseline) in are shown separately for the Social and Nonsocial conditions (Mean ± SEM across participants). L.TPJ, left temporoparietal junction; R.TPJ, right temporoparietal junction; L.TP, left temporal pole; R.TP, right temporal pole; dmpFC, dorsomedial prefrontal cortex; and PCC, posterior cingulate cortex.
