# Supplementary material for: Neural mechanisms underlying reward processing and social cognition: A replication study with a Japanese sample
Source: PLoS One. 2025 Oct 22;20(10):e0328424. doi: 10.1371/journal.pone.0328424 (PMC12543148; doi:10.1371/journal.pone.0328424)
Supplement: S2 Fig — (PDF) [file pone.0328424.s002.pdf]

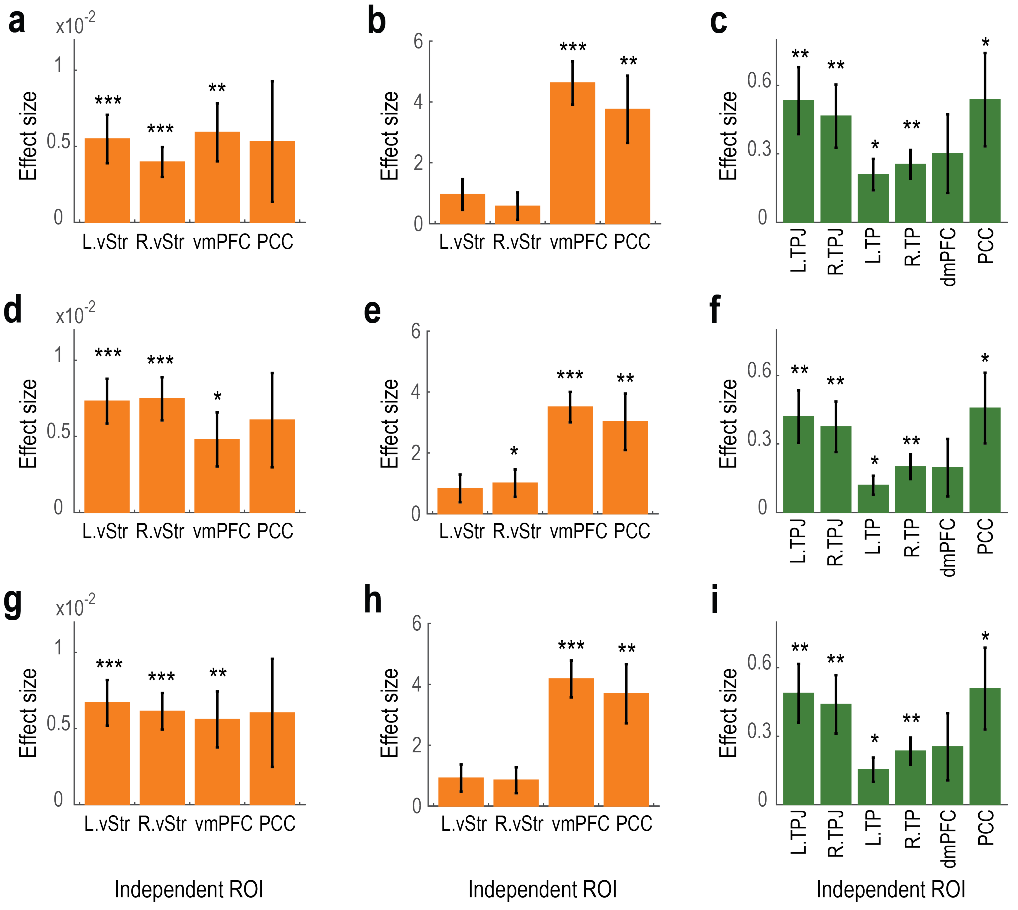

Figure S2: Supplementary analysis on the neuroimaging data II.

(a) Independent ROI analysis of reward expectation in the MID task (smoothing kernel FWHM = 4 mm and ROI sphere = 5 mm). Bar plots show the effect sizes of reward expectation (Mean ± SEM across participants) in independently defined regions of interest. L.vStr, left ventral striatum; R.vStr, right ventral striatum; vmPFC, ventromedial prefrontal cortex; and PCC, posterior cingulate cortex. ***$p < 0.001$ and **$p < 0.01$.

(b) Independent ROI analysis of reward outcome in the MID task (smoothing kernel FWHM = 4 mm and ROI sphere = 5 mm).

(c) Independent ROI analysis of social cognition in the ToM task (smoothing kernel FWHM = 4 mm and ROI sphere = 5 mm). L.TPJ, left temporoparietal junction; R.TPJ, right temporoparietal junction; L.TP, left temporal pole; R.TP, right temporal pole; and dmpFC, dorsomedial prefrontal cortex. *$p < 0.05$.

(d) Independent ROI analysis of reward expectation in the MID task (smoothing kernel

FWHM = 4 mm and ROI sphere = 10 mm).

(e) Independent ROI analysis of reward outcome in the MID task (smoothing kernel FWHM = 4 mm and ROI sphere = 10 mm).

(f) Independent ROI analysis of social cognition in the ToM task (smoothing kernel FWHM = 4 mm and ROI sphere = 10 mm).

(g) Independent ROI analysis of reward expectation in the MID task (smoothing kernel FWHM = 8 mm and ROI sphere = 5 mm).

(h) Independent ROI analysis of reward outcome in the MID task (smoothing kernel FWHM = 8 mm and ROI sphere = 5 mm).

(i) Independent ROI analysis of social cognition in the ToM task (smoothing kernel FWHM = 8 mm and ROI sphere = 5 mm).
