# Supplementary material for: Neural mechanisms underlying reward processing and social cognition: A replication study with a Japanese sample
Source: PLoS One. 2025 Oct 22;20(10):e0328424. doi: 10.1371/journal.pone.0328424 (PMC12543148; doi:10.1371/journal.pone.0328424)
Supplement: S1 Table — (PDF) [file pone.0328424.s003.pdf]

Table S1. Brain areas exhibiting significant changes in the BOLD signal associated with reward processing in the Monetary Incentive Delay task.

Contrast	Region	Hemi	x	y	z	t-statistic	*p*-value	Voxels
Reward expectation	Large cluster including ventral striatum	L/R	30	-7	-10	7.33	0.000	9961
	Temporoparietal junction	L	-54	-49	41	6.41	0.000	228
Reward outcome	Large cluster including dorsal striatum and vmPFC	L/R	0	8	14	14.07	0.000	5429
	Primary motor cortex	L/R	0	-28	68	8.80	0.000	1119
	Dorsolateral prefrontal cortex	R	-21	38	50	8.45	0.000	323

Activated clusters observed in the whole-brain analysis ($P < 0.05$ cluster-level corrected) of fMRI. vmPFC: ventromedial prefrontal cortex. The unthresholded activation maps are available at NeuroVault (https://neurovault.org/images/901831/ and https://neurovault.org/images/901832/).
