# Supplementary material for: Neural mechanisms underlying reward processing and social cognition: A replication study with a Japanese sample
Source: PLoS One. 2025 Oct 22;20(10):e0328424. doi: 10.1371/journal.pone.0328424 (PMC12543148; doi:10.1371/journal.pone.0328424)
Supplement: S2 Table — (PDF) [file pone.0328424.s004.pdf]

Table S2. Brain areas exhibiting significant changes in the BOLD signal associated with social cognition in the Theory of Mind task.

Contrast	Region	Hemi	x	y	z	t-statistic	*p*-value	Voxels
Social cognition	Temporal pole	R	54	8	-19	6.32	0.000	237
	Primary visual cortex	L/R	3	-76	2	5.48	0.000	131
	Temporoparietal junction	R	54	-46	23	4.94	0.000	138

Activated clusters observed in the whole-brain analysis ($P < 0.05$ cluster-level corrected) of fMRI. The unthresholded activation map is available at NeuroVault (https://neurovault.org/images/901812/).
